# Reproducible stability of verbal and spatial functions along the menstrual cycle

**DOI:** 10.1038/s41386-023-01789-9

**Published:** 2024-01-24

**Authors:** Belinda Pletzer, Hannah Bodenbach, Marcel Hoehn, Linda Hajdari, Tobias Hausinger, Isabel Noachtar, Adriene M. Beltz

**Affiliations:** 1 Department of Psychology, University of Salzburg, Salzburg, Austria; 2 Centre for Cognitive Neuroscience, University of Salzburg, Salzburg, Austria; 3 Department of Psychology, University of Michigan, Ann Arbor, MI USA

**Keywords:** Human behaviour, Predictive markers

## Abstract

Recent studies have reported brain changes in response to ovarian hormonal fluctuations along the menstrual cycle. However, it remains unclear, whether these brain changes are of an adaptive nature or whether they are linked to changes in behavior along the menstrual cycle, particularly with respect to cognitive performance. To address this knowledge gap, we report results from 3 well-powered behavioral studies with different task designs, leveraging the advantages of each design type. In all three studies we assessed whether verbal or spatial performance (i) differed between cycle phases, (ii) were related to estradiol and / or progesterone levels and (iii) were moderated by individual hormone sensitivity as estimated by premenstrual symptoms. Overall, results of all three studies point towards a null effect of menstrual cycle phase and – to a lesser extent – ovarian hormones on verbal and spatial performance and provided no evidence for a moderation of this effect by individual hormone sensitivity. We conclude that there is substantial consistency in verbal and spatial performance across the menstrual cycle, and that future studies of intra-individual variation are needed.

## Introduction

Verbal and spatial skills encompass a variety of cognitive functions related to the encoding, retrieval and manipulation of words and visuo-spatial relationships, respectively, that help people navigate their social and material environment in everyday life. Tasks assessing specific verbal and spatial functions, like verbal fluency or verbal memory, as well as mental rotation and navigation, produce robust and reliable sex/gender differences [1–4] and have thus been studied extensively with respect to potential masculinizing and feminizing influences of the social environment on the one hand (e.g. [5]), and sex hormones on the other hand [6]. Accordingly, verbal and spatial functions have also been repeatedly studied along the menstrual cycle (for reviews see [7, 8]), with research questions following the general rationale that a “feminization” of cognitive functions would occur during phases with higher levels of ovarian hormones. Accordingly, improved verbal performance and impaired spatial performance have been hypothesized during (i) the peri-ovulatory phase, when estradiol levels peak and (ii) the mid-luteal cycle phase, when progesterone levels peak and estradiol levels are elevated in comparison to the early follicular phase, when both hormones are at their lowest. Some studies argue that these fluctuations in cognitive functions may affect the size of sex/gender differences (e.g. [9]).

While some studies provide support for this hypothesis [10–21] (but see refs. [22–31]), there is huge variability in findings across studies, such that systematic review approaches and meta-analyses argue for a null effect of menstrual cycle phase on verbal and spatial functions [7]. However, whether the inconsistencies in the current literature reflect a true null effect or are the result of methodological issues like small sample sizes or inconsistent definitions of menstrual cycle phases remains undecided. Large-scale behavioral approaches including well-powered samples with rigorous methodology are still lacking.

Regarding designs, longitudinal studies are often chosen because they have the advantage of increased power and provide some validation for menstrual cycle phases by comparing hormone levels across phases within the same participant [32]. However, verbal and spatial tasks are subject to strong learning effects. Even when test sessions are counterbalanced, the performance increase due to repeated measurements may mask menstrual cycle dependent changes, especially when they are subtle. Cross-sectional study designs on the other hand require large sample sizes to capture menstrual cycle effects of even moderate effect size and hormone levels are usually assessed without a reference point to validate the respective cycle phase. Accordingly, results from both study types are necessary to draw conclusions on menstrual cycle effects in verbal and spatial functions.

Regarding menstrual cycle phases, the original hypotheses regarding hormonally mediated changes in cognitive functions were focused on estrogenic actions and thus call for study designs including the peri-ovulatory phase when estradiol levels peak and progesterone levels are still low. However, the estradiol peak is notoriously hard to capture resulting in high rates of data exclusions, which has prompted researchers to center their hypotheses around the luteal cycle phase. However, estradiol and progesterone have opposite effects on various neurophysiological processes, including neurogenesis [33], synaptogenesis [34] and a plethora of neurotransmitter systems [35] and also demonstrate interactive effects, e.g., by estrogen priming of progesterone receptor synthesis [36]. Accordingly, significant findings during the luteal phase cannot be attributed exclusively to estradiol, progesterone, or interactive actions, and null findings do not necessarily preclude ovarian hormone effects because progesterone and estradiol actions may cancel each other out. Accordingly, menstrual cycle designs call for the inclusion of at least 3 cycle phases in order to model the different hormonal milieus.

Furthermore, people may differ in their individual sensitivity to ovarian hormones due to differences in their neurophysiology including neurotransmitter levels (e.g. [37]) or the genetic make-up of ovarian hormone receptors (e.g. ref. [38]). For instance, a heightened sensitivity to ovarian hormonal fluctuations is thought to underlie mood disturbances in the days leading up to menstruation, i.e., premenstrual symptoms (e.g. ref. [39]). It has also been demonstrated that women with stronger premenstrual symptoms are at higher risk for developing postpartum depression [40], which in turn has been linked to the likelihood of developing depressive symptoms during hormonal contraceptive use [41]. Accordingly, there seems to be a pattern of individualized vulnerability to hormonal changes across the lifespan. However, while such individualized vulnerability and variability in the extent of changes have been described for emotional changes in response to ovarian hormones (e.g. ref. [42]), individual differences in cognitive changes along the menstrual cycle have not been taken into account. Accordingly, one explanation for inconsistencies among previous findings, is that samples differ in their sensitivity to hormonal fluctuations. One possibility to address this issue is by including the severity of premenstrual symptoms as an indirect measure of individual hormone sensitivity.

Finally, neuroimaging studies convincingly demonstrate changes in brain structure, function and connectivity along the menstrual cycle in relation to both spatial and verbal tasks [23, 28, 31, 43, 44]. However, the majority of neuroimaging studies demonstrate changes in neuronal processing that are not accompanied by changes in behavioral performance [23, 31, 43, 44]. These changes may thus reflect compensatory mechanisms (e.g., an adaptive shift in cognitive strategy or processing style) to uphold task performance during different hormonal milieus [45]. Such strategy shifts are not captured by overall performance levels and require creative task designs in which different conditions reflect different processing styles. Strategy shifts along the menstrual cycle have previously been reported for navigation and verbal fluency tasks and are thought to underlie some portion of the average gender difference in mental rotations skills [30, 46].

To fill these notable knowledge gaps surrounding the role of ovarian hormones in verbal and spatial skills, in the current manuscript we present data from 3 large scale behavioral menstrual cycle studies with different task designs, leveraging the advantages of each design type. All three studies included (i) a verbal task (verbal memory / verbal fluency), and (ii) a spatial mental rotation task. Studies 2 and 3 additionally included a spatial navigation task.

Study 1 utilized an intensive longitudinal design in which female participants provided saliva for ovarian hormone assays and completed cognitive tests for up to 80 days, that is, over 2-3 menstrual cycles. While this design calls for economically reasonable task versions that do not allow the tracking of strategy shifts, daily hormonal measurements and the averaging of multiple menstrual cycles within the same participant allow for near-perfect characterization of menstrual cycle phases.

Study 2 utilized a classical longitudinal design including all 3 cycle phases of interest (menses, per-ovulatory and luteal) in a counterbalanced fashion. In this study, strategy shifts were accounted for in all tasks employed and the large sample size and within-subject approach allowed us to achieve maximal statistical power. However, in both Study 1 and Study 2, learning effects across repeated measurements represent a possible confound.

Accordingly, Study 3 utilized a cross-sectional design including one measurement time point per participant, scheduled in one of the three cycle phases of interest (menses, peri-ovulatory, luteal). While the power for this study was slightly lower than for Study 2, learning effects do not represent an issue and cognitive processing styles were accounted for by utilizing the same tasks as in Study 2.

In all three studies, we first assessed whether verbal performance improved, and spatial performance was impaired during either the peri-ovulatory or luteal phase compared to menses. Second, we assessed whether verbal or spatial performance were related to estradiol andor progesterone levels. Third we assessed whether any shifts in cognitive processing styles could be observed along the menstrual cycle or in relation to estradiol and/or progesterone based on the data from Studies 2 and 3. Finally, as a marker of individual differences in hormone sensitivities, we assessed whether PMS symptoms moderated verbal and spatial performance changes along the menstrual cycle.

## Materials & methods

### Participants

All studies included healthy female participants, aged 18 to 35 years, who had no psychological, neurological or endocrinological disorders and did not take any medication and have not used hormonal birth control in the past 6 months. The most important inclusion criterion was a regular menstrual cycle of 21-35 days in length and a variation between individual cycles of less than 7 days. Before entering the respective study, participants provided cycle data of at least three menstrual cycles from either a menstrual cycle app or calendar. Table 1 provides an overview of the demographics and menstrual cycle characteristics of the participants included in each study.

**Table 1:** Power considerations, demographic data and menstrual cycle characteristics for each sample.

	Study 1	Study 2	Study 3
Design	Intensive longitudinal	Classical longitudinal	Cross-sectional
*Sample size*			
Recruited	40	75	186
Exclusions	1 (2.5%)	10 (13%)	33 (18%)
Final sample size	39	65^a^	153
*Demographic Data*			
Age	25.74 ± 4.90	24.03 ± 3.89	23.57 ± 3.86
Cognitive Ability [IQ-scaled]	106.53 ± 8.94	106.78 ± 10.35	106.81 ± 10.57
Higher Education	92%	98%	94%
Employment	69%	42%	42%
Relationship	41%	57%	51%
Heterosexual	80%	85%	80%
Gender Identity: Woman	100%	100%	97%
Nulliparous	90%	98%	96%
Prior use of HC	82%	57%	60%
Smokers	8%	6%	11%
PMS Diagnosis	0%	0%	0.7%
PMS (PMDD) acc. to PSST	30% (15%)	39% (15%)	36% (11%)
*Menstrual cycle (MC) characteristic*			
MC length	28.17 ± 2.67	28.80 ± 2.26	29.05 ± 2.80
MC variability	1.92 ± 1.77	2.30 ± 1.39	2.37 ± 1.70
*MC days (forward counting)*			
Menses	3.01 ± 0.24	4.06 ± 1.16	4.22 ± 1.25
Peri-ovulatory	13.72 ± 3.03	14.54 ± 2.55	13.61 ± 2.46
Luteal	21.86 ± 2.83	22.65 ± 2.60	22.12 ± 3.08
*MC days (backwards counting)*			
Menses	−25.88 ± 2.77	−25.27 ± 3.02	−25.69 ± 3.86
Peri-ovulatory	−15.11 ± 1.80	−14.90 ± 2.00	−15.17 ± 2.49
Luteal	−6.97 ± 1.87	−6.27 ± 2.99	−7.02 ± 2.27

Please note that the first session of Sample 2 was also used as part of Sample 3.

*PMS* Premenstrual syndrome, *PMDD* Premenstrual dysphoric disorder, *PSST* Premenstrual Symptom Screening Tool.

^a^Please note that 18 participants in this sample only had 2 sessions, because one session had to be excluded due to inconsistencies between estimated cycle phase and hormone values.

The recruited sample size was based on a priori power estimations to detect small to moderate effects in Studies 1 and 2 and moderate effects in Study 3 using GPower 3.1.9.7 (compare Supplementary Material). We excluded participants with a cycle length of more than 35 days during the study, resulting in anovulatory cycles without a progesterone increase during the luteal cycle phase (Study 1: *n* = 1, Study 2: *n* = 5, Study 3: *n* = 7), as well as participants with inconsistencies between the calculated and actual cycle phases based on backwards counting of cycle days from the onset of next menses and hormone levels (Study 2: *n* = 5, Study 3: *n* = 26) (compare [32]).

### Ethics statement

For each study, participants provided informed written consent to participate in the study. All methods conform to the Declaration of Helsinki and were approved by the University of Salzburg’s ethics committee.

### Procedures and Tasks

For each study, participants competed a Screening at the beginning of the first test session during which they provided demographic information, completed the Screening version of the Advanced Progressive Matrices (APM [47]), to estimate general cognitive ability, as well as the Premenstrual Symptom Screening Tool (PSST, [48]) to estimate their hormone sensitivity. The PSST consists of 14 items assessing premenstrual symptoms, as well as 5 items assessing their impact on everyday life. The PSST score was calculated by averaging responses to the 14 symptom-related items, while impact on everyday life was only used to assess whether participants meet the diagnostic criteria for PMS/PMDD according to the criteria outlined by Steiner and colleagues [48] (compare Table 1).

For Study 1, participants completed a verbal memory and mental rotation task via an online diary for 70-80 days. The verbal memory task [49] consisted of five word-pairs which participants were instructed to memorize at the beginning of each diary. At the end of each diary, they were prompted with one word from each pair and had to provide the second one. The mental rotation task consisted of 20 pairs of three-dimensional figures from the Peters and Batista (2008) stimulus library [50], for which participants had to decide whether they were the same, but rotated, or different. Participants were allowed to miss individual days in between diary entries, for which data were imputed as mean values of the previous and following day. Participants were instructed not to miss more than 5 days between diary entries. On average 4 data points per participant (SD = 3 data points) were imputed. Data from the first 5 days were discarded to account for initial training effects, accordingly 45-82 responses per participant (on average 70 responses) were included in the analyses. For each day, separate task versions were created and sent out to the participants via an online link at 5 pm in the afternoon. Participants had until 10 pm to complete the diary. The timeframe was chosen to control for diurnal fluctuations in hormone levels. Participants provided one saliva sample before they started the diary and one saliva sample after completing the diary, which were frozen at −20° in their home freezer until picked up by our lab technician. Participants completed ovulation tests starting 5 days before the expected ovulation and recorded positive ovulation tests (Pregnafix®) as well as menses onsets in the online diary. Saliva tubes and ovulation tests were delivered to their home prior to the study. Hormone levels were analyzed for each test day and smoothed using a moving average over 5 consecutive days (compare e.g. ref. [51]), For cycle comparisons, performance data were selected from (i) cycle day 3 (menses session), (ii) local estradiol maxima around ovulation (peri-ovulatory sessions), (iii) global progesterone maxima per cycle (mid-luteal sessions). Depending on cycle lengths sessions from 2-3 cycles per participant were included. For hormonal associations all test days per participants were included.

For Study 2, participants completed 3 sessions in a computer laboratory at the University of Salzburg. Test sessions were time-locked to menses, peri-ovulatory and mid-luteal cycle phase, with a counter-balanced order. Menses sessions were scheduled 3-6 days after onset of menses, peri-ovulatory sessions were scheduled 2-3 days before the expected ovulation date and mid-luteal sessions were scheduled 7 days before the expected onset of next menses. The average menstrual cycle length was determined based on the menses onsets of the last 3 menstrual cycles and added to the onset of the last menses to determine the expected onset of next menses. Ovulation was expected 14 days before the expected onset of menses and was confirmed via ovulation tests (Pregnafix®). Peri-ovulatory sessions were included in the analyses if (i) backwards counting confirmed a cycle day between −17 and −12 and (ii) estradiol levels were elevated compared to the menses session. Luteal sessions were included in the analyses if (i) backwards counting confirmed a cycle day between −11 and −4 and (ii) progesterone levels were elevated compared to the menses session. Based on these criteria, the peri-ovulatory session had to be excluded in 13 participants and the luteal session in 5 participants. Three versions of a cognitive test battery including (i) a phonemic and semantic verbal fluency task, (ii) a mental rotation task controlling for rotation angle, (iii) a navigation task controlling for navigation strategy were created and participants completed one version per session, with a counterbalanced order. In the verbal fluency task [30], participants had to produce as many words as possible within one minute for each of three letters and three semantic categories. In the mental rotation task, participants were provided with 30 pairs of three-dimensional figures from the Ganis & Kievit (2015) stimulus library [52] and had to decide whether they were the same, but rotated, or different. In the navigation task [53], participants completed 20 levels, in which they had to find a target location in a virtual environment as quickly as possible and provide their orientation according to cardinal directions (north, south, east, west) at the end. A total of 5 saliva samples were collected throughout the session for hormone assessments.

For Study 3, participants completed one session in a computer laboratory at the University of Salzburg with the same set up as in Study 2. Depending on the time since their last menses onset, test sessions were scheduled either during menses (cycle days 3-6), during the peri-ovulatory phase (2-3 days before the expected ovulation), or during the mid-luteal phase (7 days before the expected onset of next menses). The timing of sessions was determined as in Study 2, but confirmation of individual cycle phases could not rely on comparison of hormone levels with the respective menses session of the participants and was thus only determined by backwards counting of cycle days from the reported onset of next menses. Details for each task can be found in the Supplementary Material.

### Hormone analysis

Saliva samples were centrifuged twice for 15 and 10 min respectively at 3000 rpm to remove solid particles. Saliva samples from the same test session were pooled to obtain an average hormone assessment across the test session. Estradiol and progesterone levels were assessed in duplicate from pooled samples using Salimetrics salivary ELISA kits (www.salimetrics.com).

### Statistical analysis

Statistical analysis was carried out using R 4.2.3. For outlier correction, refer to Supplementary Matreial. Performance measures were evaluated in the context of a linear mixed effects model using the *lme* function of the *lme4* package [54] (for details see Supplementary Material). *P*-values were FDR corrected for multiple comparisons in each study. Frequentist statistics were accompanied by Bayesian analyses using the *lmBF* function of the *BayesFactor* package [55]. Bayes factors provide information on the relative likelihood (via odds ratios) of two statistical models, given the data: a null model (here excluding cycle phase) and an alternative model (here, including cycle phase) (for details see Supplementary Material).

## Results

### Hormone levels

**Fig. 1:**
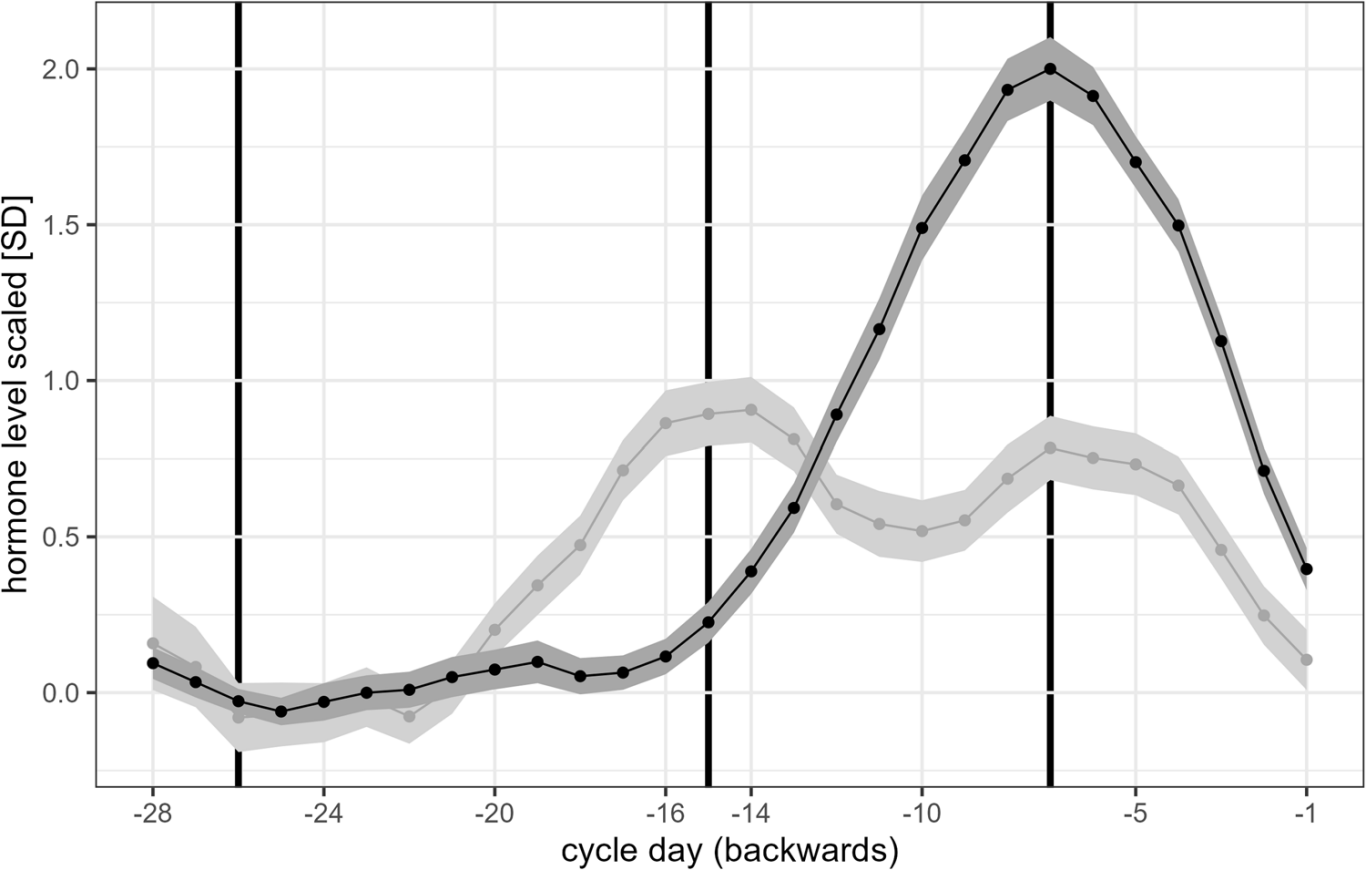
Individually standardized salivary estradiol (light gray) and progesterone (dark gray) values averaged across menstrual cycles in Study 1. Values were centered such that a value of 0 corresponds to the average hormone levels during menses. Estradiol peaked around 14-16 days before the onset of next menses and progesterone levels 7 days before the onset of next menses.

Figure 1 displays daily salivary hormone levels across the menstrual cycle in Study 1 and the time points selected for cognitive assessment. In all three studies, estradiol levels were significantly elevated during the peri-ovulatory phase compared to menses and progesterone levels were significantly elevated during the luteal cycle phase compared to menses and the peri-ovulatory phase (compare Table 2).

**Table 2:** Salivary hormone values (in pg/ml) per cycle phase in each study.

Study1			Study2		Study3	
Estradiol		Progesterone	Estradiol	Progesterone	Estradiol	Progesterone
Menses	0.90 ± 0.40	37.28 ± 31.72	0.65 ± 0.47	34.74 ± 27.76	0.70 ± 0.54	29.22 ± 26.56
Ovulatory	1.25 ± 0.53^***^	53.24 ± 50.44	1.10 ± 0.63^***^	44.70 ± 29.55	1.03 ± 0.45^***^	30.81 ± 23.33
Luteal	1.13 ± 0.49^***^	151.61 ± 92.84^###^	1.02 ± 0.51^***^	155.56 ± 98.43^###^	0.92 ± 0.57	123.97 ± 68.92^###^

^***^p < 0.001, significantly elevated compared to menses; ^###^*p* < 0.001, significantly elevated compared to menses and periovulatory phase.

### Verbal and spatial performance

**Fig. 2:**
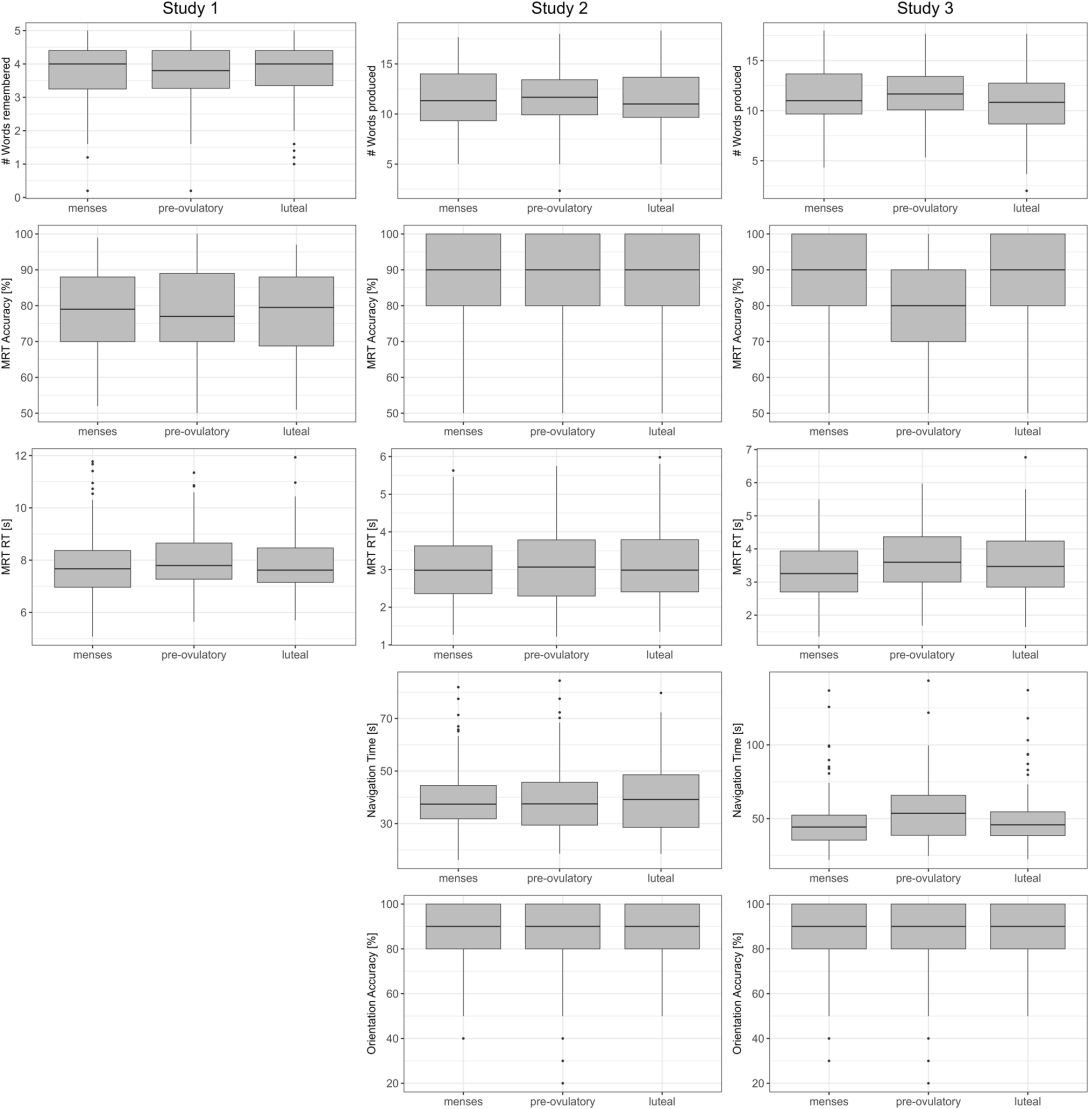
Verbal and spatial performance along the menstrual cycle in 3 studies. Study 1 used an intensive longitudinal design with daily measurements to precisely time the cycle phases. Study 2 used a classical longitudinal design with 3 time points to relatively estimate the cycle phases. Study 3 used a between-subjects design with only one time point. Study 1 used a verbal memory task (top row of left column) and a mental rotation task (MRT; middle and bottom rows of left column) based on the Peters and Batista stimulus library. Studies 2 and 3 used a verbal fluency task (top row of middle and right columns, respectively), a mental rotation task based on the Ganis and Kievit stimulus library (middle rows of middle and right columns, respectively, as well as a navigation task (bottom two rows of middle and right columns, respectively).

Results of menstrual cycle analyses on verbal and spatial performance are summarized in Supplementary Tables 1 and 2 and displayed in Fig. 2. There were no significant differences in performance between cycle phases in any task in any study (all η_p_² ˂ 0.05, all F ˂ 3.85, all p_FDR_ ˃ 0.12, Supplementary Table 2). In Studies 1 and 2, Bayes factors were generally in support of a null effect, given that the models without cycle phase were 8 to 30 times more likely than the models including cycle phase (Supplementary Table 2). However, in Study 3 for the verbal fluency and navigation tasks the models without cycle phase were only about 2 times more likely than the models including cycle phase, while for the mental rotation task, Bayes factors suggest that the models including cycle phase are about as likely than the models without cycle phase.

### Verbal and spatial processing styles

Menstrual cycle effects on verbal fluency were not significantly moderated by task condition (phonemic vs. semantic) and the number of clusters did not differ across cycle phases (all η_p_² ˂ 0.02, all F ˂ 2.32, all p_FDR_ ˃ 0.60, Supplementary Table 2). Menstrual cycle effects on mental rotation and navigation performance were not significantly moderated by rotation angle and navigation strategy respectively (all η_p_² ˂ 0.01, all F ˂ 2.11, all p_FDR_ ˃ 0.73, Supplementary Table 2). Again, Bayesian analyses were in support of a null effect, given that in both studies the models without the strategy * cycle interaction were at least 5 times more likely than the models including the interaction.

### Hormonal associations

No significant associations to estradiol or progesterone emerged in the frequentist statistics (all η_p_² ˂ 0.02, all F ˂ 5.39, all p_FDR_ ˃ 0.06). For the majority of associations frequentist statistics were backed up by Bayesian analyses indicating about 3 times higher likelihood for the models without estradiol and/or progesterone or their interaction. However, some of the Bayesian analyses were inconclusive, suggesting that the model including the hormone levels was just as likely as the model without the hormone levels (BF_01_ ~ 1, for details see Supplementary Table 3).

### Moderation by PMS symptomatology

**Fig. 3:**
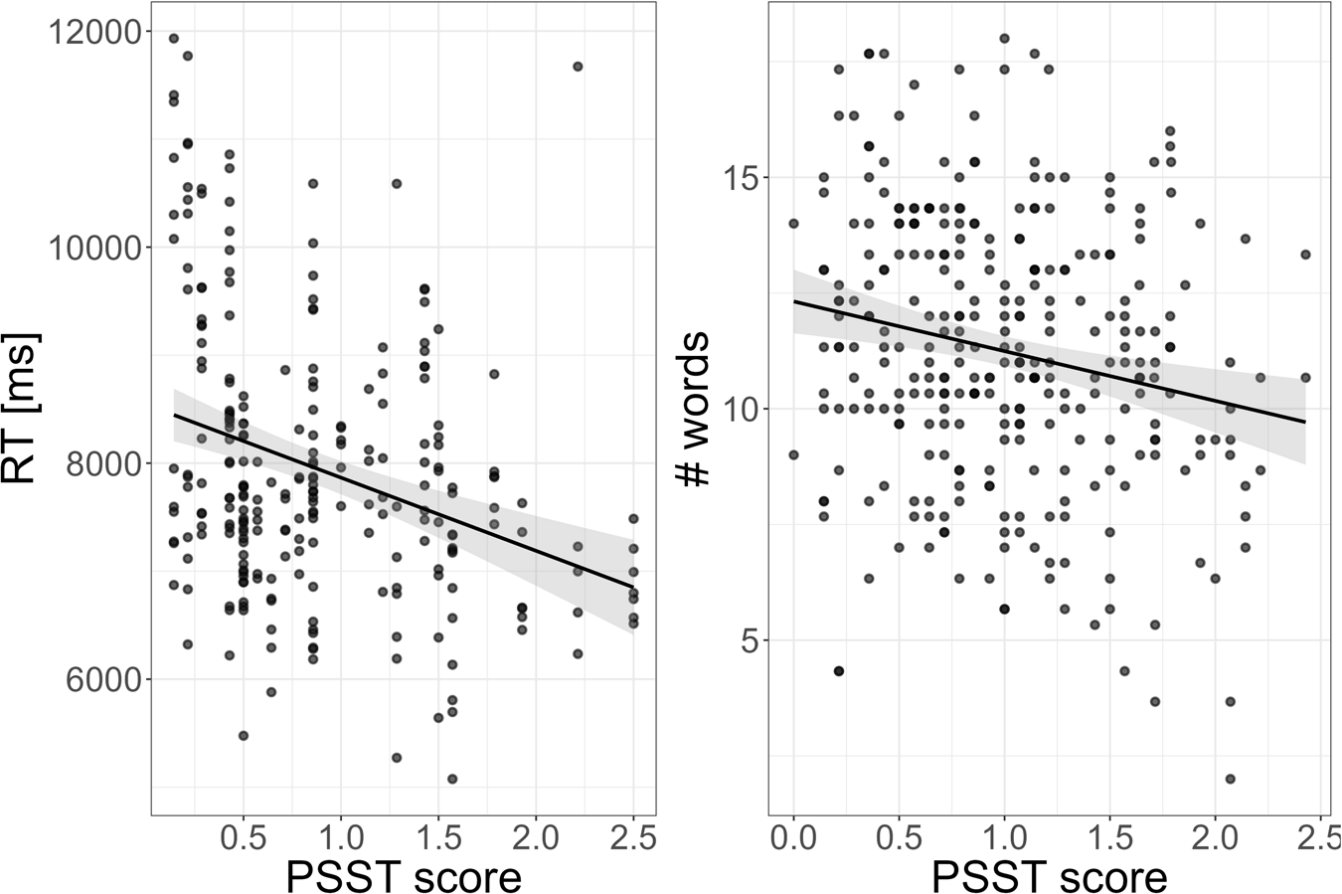
Association between premenstrual symptoms (PMS) and performance measures. Women with stronger PMS symptoms reacted faster in the mental rotation task (Study 1) and produced less words in the verbal fluency task (Study 3). The Premenstrual symptom screening tool (PSST) assesses 14 PMS symptoms on a scale from 0 (not at all) to 3 (very strong). PSST scores represent the average symptom strength over all symptoms.

The menstrual cycle effect was not moderated by PMS symptomatology in any measure of verbal or spatial performance in any study (all η_p_² ˂ 0.04, all F ˂ 2.83, all p_FDR_ > 0.37; Supplementary Table 4). There was also no significant main effect of PMS symptomatology on verbal or spatial performance in any study (all η_p_² ˂ 0.07, all F ˂ 3.76, all p_FDR_ > 0.34). With two exceptions, Bayesian analyses suggest that the models without the moderating interaction term are 3 to 30 times more likely than the models including the moderating interaction term. However, in the majority of analyses the models including the main effect of PMS were about as likely as the models without the main effect of PMS. Bayes factors even suggest that for mental rotation RT in Study 1, the number of words produced in the verbal fluency task and navigation time in Study 3, an association to PMS symptomatology is 3 and 16 times more likely than no association (Fig. 3). Women with stronger PMS symptoms reacted faster in the mental rotation task and produced less words in the verbal fluency task irrespective of their cycle phase.

## Discussion

The present manuscript describes three studies, all designed to assess menstrual cycle related changes in verbal and spatial performance, as well as a moderation by PMS symptomatology. The three studies were designed to balance considerations regarding menstrual cycle control, statistical power, and training effects on the cognitive tasks. Study 1 used an intensive longitudinal design with daily hormone measurements – it thus provided near-perfect menstrual cycle control, adequate statistical power for the cycle comparisons and high power for hormonal associations but had potentially substantial training effects due to frequent repeated assessments. Study 2 used a classic longitudinal design with 3 test sessions – it thus has good menstrual cycle control and the highest statistical power for cycle comparisons, but potential training effects. Study 1 used a cross-sectional design with three groups of women in different cycle phases – it had the poorest menstrual cycle control and the lowest power, but training effects were not present. These differences in internal validity were also reflected in exclusion rates due to anovulatory cycles or inconsistencies between estimated cycle phases and hormone levels, which were very low in Study 1 and highest in Study 3 (compare Table 1).

Overall, results of all three studies point towards a null effect of menstrual cycle phase and – to a lesser extent – ovarian hormones on verbal and spatial performance and provided no evidence for a moderation of this effect by individual hormone sensitivity as estimated by PMS symptom strength. This is in contrast to some previous studies demonstrating menstrual cycle related changes in verbal tasks (e.g. refs. [15–17, 21]), and reduced spatial performance during high estrogen cycle phases [10–14, 16, 18–20]. However, the majority of previous studies did not observe menstrual cycle related differences in verbal performance [11, 12, 20, 22–25, 27, 29–31] and most studies also found no difference in spatial performance between menses and the luteal cycle phase [15, 17, 22, 24, 27, 30, 31, 43, 56, 57]. Together, this body of work likely reflects very small effects of ovarian hormones on cognition. Given that even repeating a task for a second or third time masks those differences related to hormonal milieu, these small effects likely have little practical relevance in everyday life – in the context of the multiple biopsychosocial influences on spatial skills. They may however become potentiated for some people or in some contexts. For instance, a recent study suggests that estradiol reduces the enhancing effects of acute stress on mental rotation abilities [58]. While about a third of subjects in our studies reported mild to moderate chronic stressors, like examinations, work or relationship problems, no acute stressor was implemented during the experimental sessions. It is possible, that more noticeable reductions in spatial performance during the peri-ovulatory phase may occur in acute stress situations. Future work using intensive longitudinal designs would have the potential to estimate these intra-individual variations, e.g., via random slopes analyses. In summary, the behavioral evidence obtained in our studies points towards relative cognitive stability over the menstrual cycle. This result puts recent neuroimaging evidence on menstrual cycle related changes in brain activation and connectivity during spatial and verbal tasks into perspective (e.g., refs. [23, 31, 43]). Our data suggest that brain changes along the menstrual cycle are likely adaptive in nature with little to no global effect on performance measures. Of course, larger and more internally valid neuroimaging studies are also needed before findings are generalized more broadly, though.

This null finding regarding cognitive performance appears to be largely irrespective of whether or not women show high or low hormonal sensitivity as reflected in PMS symptoms. In neither study were menstrual cycle differences moderated by PMS symptoms with Bayesian analyses providing substantial support for this conclusion in the majority of analyses. However, results remain inconclusive regarding an association of PMS with cognitive performance irrespective of cycle phase. Regarding mental rotation, a trend towards faster responses in women with higher PMS symptoms which may reflect more impulsive decision making in women with higher PMS symptoms. Regarding verbal fluency, a trend towards lower performance in women with higher PMS symptoms was observed. This result is in accordance with some previous studies suggesting working memory deficits in women with premenstrual symptoms [59, 60]. However, the extent to which cognitive performance varies as a function of PMS symptomatology requires further investigation. For example, it remains unclear which functions are affected, whether or not the deficits vary with menstrual cycle phase and whether they can be attenuated by training. For example, a study by Schmitt et al. [61] suggests that deficits are restricted to the premenstrual phase, while our data suggest that the association to premenstrual symptoms arises irrespective of cycle phase. However, our studies did not include the premenstrual phase as they were not designed to capture main effects of PMS on cognition.

The current studies were carefully designed for capturing the main effect of cycle phase on verbal and spatial performance. Nevertheless, they are not without limitations. First, while power analyses suggest a high sensitivity for even subtle menstrual cycle effects in the longitudinal samples, the cross-sectional sample may still be underpowered to detect the most subtle differences. While we do believe that such subtle effects are likely without everyday relevance, a follow up study to dissolve the last remaining uncertainties regarding menstrual cycle effects in cross sectional samples should not only increase the sample size, but also include hormone measurements beyond the actual test session in order to obtain a frame of reference. Second, the tasks used in the current studies are only a selection of possible task options and cognitive domains and it cannot be excluded that changes in the task parameters, making the tasks even more demanding, would elicit different results. For example, a 4-response version of the mental rotation task might be more sensitive to menstrual cycle effects than the pairwise comparison used in our study [50]. In addition, the multiple task versions required for the intensive longitudinal design made it necessary to use different tasks in Study 1 than in Studies 2 and 3 (e.g., verbal memory vs. verbal fluency), which limits the comparability of results. Third, in all three samples, salivary immunoassays were used to assess hormone levels. The validity of salivary hormone assessments in predicting menstrual cycle phase, particularly for estradiol, has recently been questioned. Thus, in all three studies we combined the hormonal assessments with other methods for cycle phase validation, i.e., backwards counting and urinary ovulation kits. However, results of the first study, which uses daily hormone assessments shows that when appropriate error correction (here smoothing) is applied and the intra-individual variation rather than absolute hormone values are considered, salivary hormonal profiles do follow the expected patterns across cycle phases (compare Fig. 1).

We conclude that verbal and spatial performance remain relatively stable along the menstrual cycle in human females. Associations of verbal and spatial performance to ovarian hormones are likely weak and not moderated by individual hormone sensitivity. However, inter-individual variability in the menstrual cycle variation of cognitive performance should be further explored, capitalizing on the advantages of intensive longitudinal designs.

### Supplementary information

Supplementary Material

## Data Availability

Data and scripts are openly available at https://osf.io/bks6f/.

## References

[1] Andreano JM, Cahill L (2009). Sex influences on the neurobiology of learning and memory. Learn Mem..

[2] Nazareth A, Huang X, Voyer D, Newcombe N (2019). A meta-analysis of sex differences in human navigation skills. Psychon Bull Rev.

[3] Newcombe NS (2020). The puzzle of spatial sex differences: current status and prerequisites to solutions. Child Dev Perspect..

[4] Voyer D, Saint Aubin J, Altman K, Gallant G (2021). Sex differences in verbal working memory: a systematic review and meta-analysis. Psychol Bull..

[5] Wong WI, Yeung SP (2019). Early gender differences in spatial and social skills and their relations to play and parental socialization in children from Hong Kong. Arch Sex Behav.

[6] Hamson DK, Roes MM, Galea LA (2016). Sex hormones and cognition: neuroendocrine influences on memory and learning. Compr Physiol..

[7] Sundström Poromaa I, Gingnell M (2014). Menstrual cycle influence on cognitive function and emotion processing—from a reproductive perspective. Front Neurosci..

[8] Bernal A, Paolieri D (2022). The influence of estradiol and progesterone on neurocognition during three phases of the menstrual cycle: modulating factors. Behav Brain Res..

[9] Peragine D, Simeon-Spezzaferro C, Brown A, Gervais NJ, Hampson E, Einstein G (2020). Sex difference or hormonal difference in mental rotation? The influence of ovarian milieu. Psychoneuroendocrinology.

[10] Becker D, Creutzfeldt OD, Schwibbe M, Wuttke W (1982). Changes in physiological, EEG and psychological parameters in women during the spontaneous menstrual cycle and following oral contraceptives. Psychoneuroendocrinology.

[11] Hampson E (1990). Variations in sex-related cognitive abilities across the menstrual cycle. Brain Cogn.

[12] Phillips SM, Sherwin BB (1992). Variations in memory function and sex steroid hormones across the menstrual cycle. Psychoneuroendocrinology.

[13] Silverman I, Phillips K (1993). Effects of estrogen changes during the menstrual cycle on spatial performance. Ethol Sociobiol.

[14] Hausmann M, Slabbekoorn D, Van Goozen SH, Cohen-Kettenis PT, Güntürkün O (2000). Sex hormones affect spatial abilities during the menstrual cycle. Behav Neurosci.

[15] Rosenberg L, Park S (2002). Verbal and spatial functions across the menstrual cycle in healthy young women. Psychoneuroendocrinology.

[16] Maki PM, Rich JB, Rosenbaum RS (2002). Implicit memory varies across the menstrual cycle: estrogen effects in young women. Neuropsychologia.

[17] Solís-Ortiz S, Corsi-Cabrera M (2008). Sustained attention is favored by progesterone during early luteal phase and visuo-spatial memory by estrogens during ovulatory phase in young women. Psychoneuroendocrinology.

[18] Šimić N, Santini M (2012). Verbal and spatial functions during different phases of the menstrual cycle. Psychiatr Danub..

[19] Courvoisier DS, Renaud O, Geiser C, Paschke K, Gaudy K, Jordan K (2013). Sex hormones and mental rotation: an intensive longitudinal investigation. Horm Behav..

[20] Hampson E, Levy-Cooperman NA, Korman JM (2014). Estradiol and mental rotation: relation to dimensionality, difficulty, or angular disparity?. Horm Behav..

[21] Barel E, Krispil M, Yaari I (2019). Cognitive performance across the menstrual cycle. J Psychol Cogn..

[22] Gordon HW, Lee PA (1993). No difference in cognitive performance between phases of the menstrual cycle. Psychoneuroendocrinology.

[23] Konrad C, Engelien A, Schöning S, Zwitserlood P, Jansen A, Pletziger E (2008). The functional anatomy of semantic retrieval is influenced by gender, menstrual cycle, and sex hormones. J Neural Transm..

[24] Mordecai KL, Rubin LH, Maki PM (2008). Effects of menstrual cycle phase and oral contraceptive use on verbal memory. Horm Behav..

[25] Hatta T, Nagaya K (2009). Menstrual cycle phase effects on memory and Stroop task performance. Arch Sex Behav.

[26] Kozaki T, Yasukouchi A (2009). Sex differences on components of mental rotation at different menstrual phases. Int J Neurosci..

[27] Griksiene R, Ruksenas O (2011). Effects of hormonal contraceptives on mental rotation and verbal fluency. Psychoneuroendocrinology.

[28] Weis S, Hausmann M, Stoffers B, Sturm W (2011). Dynamic changes in functional cerebral connectivity of spatial cognition during the menstrual cycle. Hum Brain Mapp..

[29] Mihalj M, Drenjančević I, Včev A, Šumanovac A, Čavka A, Vladetić M (2014). Basic cognitive functions across the menstrual cycle in a con-trolled female cohort. Med Glas.

[30] Scheuringer A, Pletzer B (2017). Sex differences and menstrual cycle dependent changes in cognitive strategies during spatial navigation and verbal fluency. Front Psychol..

[31] Pletzer B, Harris TA, Scheuringer A, Hidalgo-Lopez E (2019). The cycling brain: menstrual cycle related fluctuations in hippocampal and fronto-striatal activation and connectivity during cognitive tasks. Neuropsychopharmacology.

[32] Hampson E (2020). A brief guide to the menstrual cycle and oral contraceptive use for researchers in behavioral endocrinology. Horm Behav..

[33] Abotalebi H, Ebrahimi B, Shahriyari R, Shafieian R (2021). Sex steroids-induced neurogenesis in adult brain: a better look at mechanisms and mediators. Horm Mol Biol Clin Investig..

[34] Litwack G., editor. Hormones and Synapse. Academic Press: Cambridge, MA; 2020.

[35] Barth C, Villringer A, Sacher J (2015). Sex hormones affect neurotransmitters and shape the adult female brain during hormonal transition periods. Front Neurosci..

[36] Ekka E, Vanderheyden I, Glorieux B, De Hertogh R (1987). Estradiol-induced progesterone receptor synthesis in normal and diabetic ovariectomized rat uterus. J Steroid Biochem..

[37] Jacobs E, D’Esposito M (2011). Estrogen shapes dopamine-dependent cognitive processes: implications for women’s health. J Neurosci..

[38] Huo L, Straub RE, Roca C, Schmidt PJ, Shi K, Vakkalanka R (2007). Risk for premenstrual dysphoric disorder is associated with genetic variation in ESR1, the estrogen receptor alpha gene. Biol Psychiatry..

[39] Comasco E, Kopp Kallner H, Bixo M, Hirschberg AL, Nyback S, De Grauw H (2021). Ulipristal acetate for treatment of premenstrual dysphoric disorder: a proof-of-concept randomized controlled trial. Am J Psychiatry..

[40] Castro RA, Pataky EA, Ehlert U (2019). Associations between premenstrual syndrome and postpartum depression: a systematic literature review. Biol Psychol..

[41] Larsen SV, Mikkelsen AP, Lidegaard Ø, Frokjaer VG (2023). Depression associated with hormonal contraceptive use as a risk indicator for postpartum depression. JAMA Psychiatry.

[42] Sundström-Poromaa I, Comasco E, Sumner R, Luders E (2020). Progesterone–Friend or foe?. Front Neuroendocrinol..

[43] Schöning S, Engelien A, Kugel H, Schäfer S, Schiffbauer H, Zwitserlood P (2007). Functional anatomy of visuo-spatial working memory during mental rotation is influenced by sex, menstrual cycle, and sex steroid hormones. Neuropsychologia.

[44] Hidalgo-Lopez E, Pletzer B (2021). Fronto-striatal changes along the menstrual cycle during working memory: effect of sex hormones on activation and connectivity patterns. Psychoneuroendocrinology.

[45] De Vries G (2004). Minireview: sex differences in adult and developing brains: compensation, compensation, compensation. Endocrinology.

[46] Hussain D, Hanafi S, Konishi K, Brake WG, Bohbot VD (2016). Modulation of spatial and response strategies by phase of the menstrual cycle in women tested in a virtual navigation task. Psychoneuroendocrinology.

[47] Raven J, Court JH. Manual for Raven’s progressive matrices and vocabulary scales. Assessment. Oxford: Oxford Psychlogists Press; 1998.

[48] Steiner M, Macdougall M, Brown E (2023). The premenstrual symptoms screening tool (PSST) for clinicians. Arch. Wom Ment Health.

[49] Kelly DP, Beltz AM (2021). Capturing fluctuations in gendered cognition with novel intensive longitudinal measures. Assessment.

[50] Peters M, Battista C (2008). Applications of mental rotation figures of the Shepard and Metzler type and description of a mental rotation stimulus library. Brain Cognition.

[51] Guevarra DA, Louis CC, Gloe LM, Block SR, Kashy DA, Klump KL (2023). Examining a window of vulnerability for affective symptoms in the mid-luteal phase of the menstrual cycle. Psychoneuroendocrinology.

[52] Ganis G, Kievit R (2015). A new set of three-dimensional shapes for investigating mental rotation processes: validation data and stimulus set. J Open Psychol Data..

[53] Harris T, Scheuringer A, Pletzer B (2019). Perspective and strategy interactively modulate sex differences in a 3D navigation task. Biol Sex Differ..

[54] Bates D, Maechler M, Bolker B, Walker S, Christensen RHB, Singmann H et al. Package ‘lme4’. 2009. http://lme4.r-forge.r-project.org/ Accessed 23 August 2023.

[55] Morey RD, Rouder JN, Jamil T, Morey MRD. Package ‘bayesfactor’. 2015. https://cran.r-project.org/web/packages/BayesFactor/BayesFactor.pdf Accessed 23 August 2023.

[56] Epting LK, Overman WH (1998). Sex-sensitive tasks in men and women: a search for performance fluctuations across the menstrual cycle. Behav Neurosci..

[57] Bianchini F, Verde P, Colangeli S, Boccia M, Strollo F, Guariglia C (2018). Effects of oral contraceptives and natural menstrual cycling on environmental learning. BMC Womens Health.

[58] Cohen A, Zemel OC, Colodner R, Abu-Shkara R, Masalha R, Mahagna L (2020). The role of endocrine stress systems and sex hormones in the enhancing effects of stress on mental rotation capabilities. Brain Sci.

[59] Yen JY, Chang SJ, Long CY, Tang TC, Chen CC, Yen CF (2012). Working memory deficit in premenstrual dysphoric disorder and its associations with difficulty in concentrating and irritability. Compr Psychiatry..

[60] Slyepchenko A, Lokuge S, Nicholls B, Steiner M, Hall GB, Soares CN (2017). Subtle persistent working memory and selective attention deficits in women with premenstrual syndrome. Psychiatry Res.

[61] Schmitt JA, Jorissen BL, Dye L, Markus CR, Deutz NE, Riedel WJ (2005). Memory function in women with premenstrual complaints and the effect of serotonergic stimulation by acute administration of an alpha-lactalbumin protein. J Psychopharmacol..

