# Supplementary material for: Reproducible stability of verbal and spatial functions along the menstrual cycle
Source: Neuropsychopharmacology. 2024 Jan 24;49(6):933–41. doi: 10.1038/s41386-023-01789-9 (PMC11039678; doi:10.1038/s41386-023-01789-9)
Supplement: Supplementary file 1 — Supplementary Material [file 41386_2023_1789_MOESM1_ESM.pdf]

Supplementary Materials for

Reproducible stability of verbal and spatial functions along the menstrual cycle

Table of contents

**Tasks**	**1**
**Power Analyses**	**3**
**Outlier Correction**	**4**
**Statistical Models**	**4**
**Supplementary Table 1: Descriptives**	**5**
**Supplementary Table 2: Results of menstrual cycle analyses**	**6**
**Supplementary Table 3: Results of hormonal association analyses**	**7**
**Supplementary Table 4: Results of PMS moderation analyses**	**8**
**References**	**9**

Tasks:

All cognitive tasks were adapted from previous studies in which they successfully elicited sex/gender differences (Kelly & Beltz, 2021; Scheuringer et al., 2017; Pletzer et al., 2019b; Harris et al., 2019). For Study 1 we selected stimuli from large stimulus data bases to create 75 different task versions. For Study 2 we selected tasks that allowed for the control of task difficulty or cognitive strategy / processing style by providing different task conditions.

Verbal Memory task (Study 1): In Study 1, participants learned 5 word-pairs at the beginning of each test session. They were given 2 seconds to memorize each word pair. After participants completed the mental rotation task and questionnaires, which took about 20 min to complete, they were prompted with one randomly selected word from each pair and had to provide the second word. The order in which word pairs were tested was also randomized. The number of correctly recalled words was recorded. In order to create 75 different versions of the verbal memory task, word pairs were translated from Kelly & Beltz (2021).

Verbal fluency task (Studies 2 & 3): In Studies 2 and 3, participants were prompted with 3 letters (phonemic task) and 3 semantic categories (semantic task). For each letter/category they had one minute to write down as many words as possible beginning with the given letter or belonging to the given semantic category. The three task versions of the phonemic task consisted of letters F-M-L, T-P-K, S-N-R. The three versions of the semantic task consisted of the categories main courses-birds-furniture, starters-mammals-beauty products, vegetables-aquatic animals-stationary. The task versions were adapted from Scheuringer et al. (2017), pre-tested in a sample of 16 participants and matched for difficulty. We recorded the number of words produced as well as the number of phonemic/semantic clusters formed. Phonemic clusters included consecutive words with the same second letter and semantic clusters consecutive words from the same semantic sub-category (compare Troyer et al., 1999).

Mental rotation task (Study 1): In Study 1, participants were presented with 20 image-pairs of 3-dimensional figures and had to decide whether the 2 images presented the same figures, albeit rotated, or whether the 2 images presented different figures within 10s. Half the figure-pairs were identical, but rotated, and half were different. In order to create 75 different versions of the mental rotation task, figures were selected from the Peters and Batista (2008) stimulus library. The number of correct responses and response times for each item were recorded.

Mental rotation task (Studies 2 and 3): In study 2, participants were presented with 30 image-pairs of 3-dimensional figures and had to decide whether the 2 images presented the same figures, albeit rotated, or whether the 2 images presented different figures within 8s. Half the figure-pairs were identical, but rotated, and half were different. In order to control for task difficulty, the stimuli were selected from the Ganis & Kievit (2015) stimulus library, which provides detailed information about rotation angle. 10 image pairs were included with a rotation angle of 50°, 10 with a rotation angle of 100° and 10 with a rotation angle of 150°. The number of correct responses and response times for each item were recorded.

Navigation task (Studies 2 and 3): The navigation task was adapted from Harris et al. (2019). Participants completed 20 levels, each consisting of a new virtual environment built as a 10x10 matrix including 10 different landmarks (trees, flowers, bushes, benches, stones, houses, churches, stairs, bridges and traffic lights) in each row and column. In each level they were given three lines of directions

leading to a final target and were instructed to reach the target as quickly as possible by moving around in the virtual environment using the arrow keys on the keyboard. In order to control for navigation strategy, directions were formulated either from an egocentric (left turn/right turn) or allocentric perspective (north, south, east, west) and referred to either Euclidian (number of blocks) or landmark information (until the tree) to describe distances. Participants could only move on to the next level if they reached the final target. Upon reaching the final target in each level participants were asked to orient themselves by identifying north. Navigation time (time to reach the final target) and orientation accuracy were recorded.

Power analyses

Power estimations were carried out in GPower 3.1.9.7. The sample size required for each study was determined a priori to provide 80% power for small to moderate effect sizes (Study 1: f = 0.20, Study 2: f = 0.15, Study 3: f = 0.25) in repeated measures ANOVA analyses assuming a correlation of 0.5 among repeated measures. For Study 1 we set the alpha error probability to 0.016 since 3 performance measures were assessed in this study and the number of measurements was set to 6, since we followed participants over approximately 2 cycles, selecting 3 phases per cycle. For Studies 2 and 3 we set the alpha error probability to 0.008, since 6 performance measures were assessed in this study. In Study 2 the number of measurements was 6 given that 2 task conditions were assessed over 3 cycle phases. In Study 3, the number of measurements included only the 2 task conditions. Using these input parameters GPower suggested a required sample size of n = 36 for Study 1, n = 71 for Study 2 and n = 180 for Study 3). An additional 4-6 participants was recruited for each study to account for drop-outs, though the final number of exclusions necessary was higher in Studies 2 and 3. Given that the analyses performed were linear mixed models rather than ANOVAs, these numbers should be considered as estimates.

Outlier correction

Conditions with accuracy measures below chance level were excluded from analyses. For outlier correction, items with reaction time (RT) measures of more than 3 standard deviations above the mean were excluded from reaction time analyses (mental rotation task, navigation task). Thus, in Study 1, 11 data points were excluded for MRT accuracy and 9 data points for MRT RT. In Study 2, 12 data points were excluded for MRT accuracy and 23 individual trials were excluded before calculating the average MRT RT. In Study 3, 20 data points were excluded for MRT accuracy and no trials were excluded before calculating the average MRT RT.

Statistical models

All linear mixed effects models included participant number (PNr) as a random factor and menstrual cycle phase (dummy coded) as a fixed effect. For longitudinal studies, session was included as a fixed effect (continuous, first session set to 0 as a reference point) to control for training effects. For Studies 2 and 3, task condition (dummy coded) and its interaction with menstrual cycle phase were included as fixed effects in order to assess menstrual cycle dependent changes in cognitive strategies. Accordingly, the following models were fitted for Study 1 (Performance ~ CyclePhase + Session + 1|PNr), Study 2 (Performance ~ CyclePhase*TaskCondition + Session + 1|PNr) and Study 3 (Performance ~ CyclePhase*TaskCondition + 1|PNr). In order to evaluate hormonal associations with performance measures, estradiol and progesterone, as well as their interaction were included as fixed effects in the models (Performance ~ Estradiol*Progesterone + Session + 1|PNr). In order to evaluate whether the menstrual cycle effect was moderated by individual differences via PMS symptoms, PMS symptoms and their interaction with cycle phase were included in the models (Performance ~ CyclePhase*PMS + Session + 1|PNr). In Study 1, hormone levels were entered once centred around the group mean and once individually centred around menses values in order to assess not only the association to absolute hormone values, but take into account individual variabilities in baseline values and assess the increase relative to the individual baseline.

In case of null effect of menstrual cycle phase, we calculated Bayes factors of a model including the cycle phase relative to the model not including cycle phase using 10.000 iterations for Monte Carlo sampling. In case of non-significant interactions, we calculated Bayes factors of a model including the interaction relative to a model including only main effects. Bayes factors (BF) quantify the relative likelihood of the observed data under two competing models. As per the function default, the prior distribution was a zero-centered Cauchy distribution with scale of 0.707. The participant number was included as random factor in the models.

	menses			peri-ovulatory			luteal		
	**mean**	**SD**	**range**	**mean**	**SD**	**range**	**mean**	**SD**	**range**
**Study 1: intensive longitudinal**									
Verbal recall [words]	3.71	1.02	0-5	3.72	0.92	0-5	3.78	0.91	1-5
MRT accuracy [%]	77.89	11.67	52-99	78.25	12.04	50-100	78.26	12.23	51-97
MRT RT [ms]	7892.34	1392.02	5076-11770	8069.36	1225.02	5642-11346	7859.22	1198.84	5696-11932
**Study 2: classic longitudinal**									
Verbal fluency [words]	11.53	3.05	5-18	11.49	2.74	2-18	11.34	2.79	5-18
Verbal fluency [clusters]	7.22	2.30	3-14	7.49	1.96	3-12	7.14	1.99	3-12
MRT accuracy [%]	86.58	13.22	50-100	85.46	13.99	50-100	86.90	12.93	50-100
MRT RT [ms]	3024.82	899.82	1267-5628	3094.92	1025.41	1220-5748	3108.33	929.97	1352-5979
Navigation time [s]	39.10	11.46	16.18-81.92	39.70	13.10	18.53-84.39	39.87	12.51	18.46-79.73
Orientation accuracy [%]	89.30	13.70	40-100	89.31	14.85	20-100	90.17	11.59	50-100
**Study 3: cross-sectional**									
Verbal fluency [words]	11.52	2.88	4-18	11.49	2.73	5-18	10.81	3.07	2-18
Verbal fluency [clusters]	7.49	2.13	2-14	7.32	1.79	4-11	6.99	2.08	2-14
MRT accuracy [%]	85.48	14.84	50-100	79.22	16.27	50-100	84.79	13.85	50-100
MRT RT [ms]	3316.18	878.49	1354-5494	3664.27	996.36	1685-5974	3517.24	936.09	1643-6766
Navigation time [s]	47.52	18.79	22.01-136.8	54.84	21.47	24.72-143.51	49.71	18.54	22.5-137.04
Orientation accuracy [%]	86.21	15.70	30-100	82.46	17.44	20-100	85.86	13.06	50-100

Supplementary Table 1: Descriptive statistics of verbal and spatial performance measures in 3 studies

	Session		Main effect of cycle phase			strategy x cycle interaction		
	η_p^2	F	η_p^2	F	BF_{01}	η_p^2	F	BF_{01}
**Study 1: intensive longitudinal**								
Verbal recall [words]	0.01	2.69	< 0.01	0.21	21.71±2.21%			
MRT accuracy [%]	0.35	114.96***	< 0.01	1.07	9.44±0.72%			
MRT RT [ms]	0.12	27.92***	0.02	1.04	8.39±1.35%			
**Study 2: classic longitudinal**								
Verbal fluency [words]	0.02	4.87	< 0.01	1.51	19.68±2.93%	0.01	1.38	5.41±2.90%
Verbal fluency [clusters]	< 0.01	< 0.01	0.02	2.32	1.46±2.34%	< 0.01	0.64	9.83±2.02%
MRT accuracy [%]	0.07	31.12***	< 0.01	1.58	10.16±1.03%	0.01	2.11	6.85±1.34%
MRT RT [ms]	0.32	212.25***	0.01	0.64	24.47±1.44%	< 0.01	0.26	104.35±1.28%
Navigation time [s]	0.37	163.64***	< 0.01	0.09	29.38±1.92%	< 0.01	0.32	13.25±2.08%
Orientation accuracy [%]	0.16	51.85***	< 0.01	0.86	13.18±4.10%	< 0.01	0.15	16.16±4.12%
**Study 3: cross-sectional**								
Verbal fluency [words]			0.02	0.94	2.25±1.09%	0.01	1.03	6.42±1.74%
Verbal fluency [clusters]			0.03	2.56	2.56±1.39%	0.01	0.96	6.61±1.82%
MRT accuracy [%]			0.05	3.84	0.63±1.46%	0.01	0.84	23.08±1.12%
MRT RT [ms]			0.03	2.24	1.49±2.98%	< 0.01	0.64	41.42±2.89%
Navigation time [s]			0.03	1.95	2.06±1.68%	< 0.01	0.10	13.11±2.67%
Orientation accuracy [%]			0.02	1.57	4.11±1.35%	0.01	0.89	7.11±1.33%

Supplementary Table 2: Results of menstrual cycle analyses. *p < 0.05, ***p < 0.001

	Estradiol			Progesterone			estradiol x prog. interaction		
	η_p^2	F	BF_{01}	η_p^2	F	BF_{01}	η_p^2	F	BF_{01}
**Study 1: intensive longitudinal (~75 days / participant)**									
Verbal recall [words]	<0.01	0.03	6.78±0.92%	<0.01	1.06	7.63±0.94%	<0.01	1.79	3.45±0.76%
MRT accuracy [%]	<0.01	0.56	10.39±2.37%	<0.01	0.17	16.75±2.54%	<0.01	3.14	4.03±1.50%
MRT RT [ms]	<0.01	3.45	3.49±2.52%	<0.01	4.03	3.77±3.17%	<0.01	4.16	1.49±4.16%
**Study 2: classic longitudinal (3 sessions per participant)**									
Verbal fluency [words]	<0.01	0.01	3.35±0.79%	0.01	1.32	1.93±0.50%	<0.01	0.70	2.68±0.39%
Verbal fluency [clusters]	<0.01	0.87	2.41±0.67%	0.01	3.13	0.95±0.47%	<0.01	0.01	3.72±0.37%
MRT accuracy [%]	<0.01	0.09	3.93±0.51%	<0.01	0.28	4.59±0.49%	<0.01	0.10	4.51±0.78%
MRT RT [ms]	<0.01	0.13	5.44±1.11%	0.02	2.33	2.44±1.03%	<0.01	0.03	7.25±0.84%
Navigation time [s]	<0.01	0.06	5.38±0.52%	0.01	0.45	6.46±0.51%	<0.01	0.13	6.55±0.41%
Orientation accuracy [%]	<0.01	2.11	1.66±0.69%	0.01	3.53	0.75±0.85%	<0.01	1.27	2.83±0.64%
**Study 3: cross-sectional (1 session per participant)**									
Verbal fluency [words]	<0.01	0.78	2.96±0.94%	0.03	3.30	0.64±1.08%	0.01	1.22	2.09±0.56%
Verbal fluency [clusters]	<0.01	1.05	2.99±0.88%	0.02	2.81	1.12±0.87%	<0.01	0.67	2.96±0.47%
MRT accuracy [%]	<0.01	<0.01	3.92±2.66%	<0.01	0.04	3.84±2.57%	<0.01	0.16	3.17±0.24%
MRT RT [ms]	<0.01	0.19	3.00±8.07%	<0.01	0.03	2.87±3.51%	<0.01	0.01	2.36±4.46%
Navigation time [s]	0.02	2.67	0.98±1.41%	<0.01	0.02	3.16±1.33%	<0.01	0.50	2.12±1.30%
Orientation accuracy [%]	<0.01	0.14	3.05±2.55%	0.01	0.48	2.26±2.74%	0.01	1.31	1.59±3.40%

Supplementary Table 3: Results of hormonal association analyses. For the majority of associations frequentist statistics were backed up by Bayesian analyses indicating about 3 times higher likelihood for the models without estradiol and/or progesterone or their interaction. However, it was unclear, whether progesterone related to verbal fluency performance and whether estradiol related to navigation performance in Studies 2 and 3.

	PMS			cycle x PMS interaction		
	**η_p^2**	**F**	**BF_{01}**	**η_p^2**	**F**	**BF_{01}**
**Study 1: intensive longitudinal**						
Verbal recall [words]	0.01	0.58	1.82±3.32%	< 0.01	0.49	10.59±6.09%
MRT accuracy [%]	0.02	0.71	1.93±3.34%	< 0.01	0.08	54.20±4.82%
MRT RT	**0.15**	**3.26**	**0.28±1.27%**	0.01	1.43	11.65±3.60%
**Study 2: classic longitudinal**						
Verbal fluency [words]	0.01	0.32	2.44±1.37%	< 0.01	0.49	12.14±6.42%
Verbal fluency [clusters]	0.01	0.46	2.93±1.83%	< 0.01	0.04	16.84±1.24%
MRT accuracy [%]	0.02	0.12	2.23±1.25%	0.01	2.65	2.59±2.92%
MRT RT [ms]	0.01	1.08	2.73±1.31%	0.01	1.31	24.22±1.24%
Navigation time [s]	0.03	3.76	1.08±1.24%	0.01	0.98	29.59±0.80%
Orientation accuracy [%]	0.03	3.29	1.47±1.53%	0.01	0.73	16.50±1.09%
**Study 3: cross-sectional**						
Verbal fluency [words]	**0.07**	**0.36**	**0.06±1.05%**	0.01	0.93	8.84±1.90%
Verbal fluency [clusters]	**0.05**	**1.03**	**0.32±1.26%**	< 0.01	0.14	17.97±0.97%
MRT accuracy [%]	0.02	3.58	1.08±0.99%	0.01	0.55	9.89±4.12%
MRT RT [ms]	0.04	0.95	0.41±0.93%	0.03	2.11	1.75±3.85%
Navigation time [s]	**0.05**	**0.95**	**0.15±0.96%**	0.04	2.83	1.02±2.88%
Orientation accuracy [%]	0.02	1.71	1.00±0.65%	< 0.01	0.09	9.14±3.57%

Supplementary Table 4: Results of PMS moderation analyses

References

Ganis G, Kievit R. A new set of three-dimensional shapes for investigating mental rotation processes: validation data and stimulus set. J. open psychol. Data. 2015;3:e3.

Harris T, Scheuringer A, Pletzer B. Perspective and strategy interactively modulate sex differences in a 3D navigation task. Biol. Sex Differ. 2019;10:1-12.

Kelly DP, Beltz AM. Capturing fluctuations in gendered cognition with novel intensive longitudinal measures. Assessment. 2021;28:1813-1827.

Pletzer B, Steinbeisser J, Van Laak L, Harris T. Beyond biological sex: interactive effects of gender role and sex hormones on spatial abilities. Front. Neurosci. 2019;13:675.

Troyer AK. Normative data for clustering and switching on verbal fluency tasks. J. Clin. Exp. Neuropsychol. 2000;22:370-378.
